# Emerging functions of tissue-resident eosinophils

**DOI:** 10.1084/jem.20221435

**Published:** 2023-06-16

**Authors:** Alessandra Gurtner, Daniel Crepaz, Isabelle C. Arnold

**Affiliations:** 1 Institute of Experimental Immunology, University of Zürich, Zürich, Switzerland

## Abstract

Eosinophils are typically considered tissue-damaging effector cells in type 2 immune-related diseases. However, they are also increasingly recognized as important modulators of various homeostatic processes, suggesting they retain the ability to adapt their function to different tissue contexts. In this review, we discuss recent progress in our understanding of eosinophil activities within tissues, with particular emphasis on the gastrointestinal tract, where a large population of these cells resides under non-inflammatory conditions. We further examine evidence of their transcriptional and functional heterogeneity and highlight environmental signals emerging as key regulators of their activities, beyond classical type 2 cytokines.

## Teaser

Arnold et al. review the pleiotropic functions of tissue-resident eosinophils and examine their contribution to immune and tissue homeostasis. They further discuss evidence of the plasticity of this lineage, highlighting environmental signals emerging as key regulators of eosinophil functional polarization.

## Introduction

Eosinophils originate in the bone marrow from common granulocyte/monocyte progenitors, and their lineage commitment is instructed by a combinatorial program of transcription factors, including high expression of GATA-1 (Yu et al., 2002; Bouffi et al., 2015; Hirasawa et al., 2002; Fulkerson, 2017). IL-5, the most eosinophil-specific cytokine, is required for the expansion and maturation of eosinophil progenitors and further supports the survival and activation of eosinophils within tissues, together with the other β common chain cytokines, GM-CSF and IL-3 (Fulkerson et al., 2014; Griseri et al., 2015; Collins et al., 1995; Rothenberg et al., 1989; Dent et al., 1990). As eosinophils exit the bone marrow, they spend only limited time in circulation (between 3 and 24 h), where they represent a minor component of leukocytes (Farahi et al., 2012). They then rapidly migrate to their tissues of residency following an eotaxin gradient in response to surface-expressed C-C chemokine receptor 3 (CCR3) binding (Mishra et al., 1999).

Small numbers of eosinophils are found in several peripheral tissues under non-inflammatory conditions, where they exhibit distinct turnover rates in response to local viability-enhancing signals and differential expression of the common γ-chain receptor (Carlens et al., 2009). In addition, a predominant eosinophil population is found in the mucosal lining of the (non-esophageal) gastrointestinal (GI) tract, where they represent 5–25% of the total leukocyte fraction. Their recruitment to the intestinal lamina propria occurs during fetal development, and their numbers are broadly maintained throughout lifetime, with cyclic variations fine-tuned by type 2 innate lymphoid cell (ILC2)–derived IL-5 in response to circadian rhythms and caloric intake (Nussbaum et al., 2013). Low frequencies of resident eosinophils are also found in the lung, thymus, adipose tissue, uterus, and mammary gland, where their numbers fluctuate according to hormonal cycles or developmental stages (Loffredo et al., 2020; Gouon-Evans and Pollard, 2001; Gouon-Evans et al., 2000; Throsby et al., 2000).

Moreover, eosinophils greatly accumulate at sites of tissue damage in response to infection or allergen exposure, where they can exert potent inflammatory effects through the release of their preformed cytotoxic granule proteins. The abnormal presence or accumulation of eosinophils in peripheral organs beyond baseline levels is commonly referred to as “eosinophilia” and is generally associated with diseases. Eosinophilia is not only a major hallmark of allergic asthma, atopic dermatitis, and chronic rhinosinusitis but is also typical of chronic inflammatory conditions of the GI tract, such as eosinophil-associated GI disorders, eosinophilic esophagitis, and inflammatory bowel diseases (Humbles et al., 2004; Jenerowicz et al., 2007; Raab et al., 1998; Blanchard et al., 2006). Many of these conditions are dramatically on the rise in industrialized countries, and their prevalence increases in developing countries in parallel to urbanization (Yang et al., 2020; Dellon et al., 2015; Freeman et al., 2021). However, a profound understanding of the complex signaling networks driving eosinophil pleiotropic functions in health and disease is still missing, therefore limiting clinical applications. As the majority of eosinophils reside in peripheral tissues under baseline conditions, it is of great interest to understand their roles and regulation at these sites.

In this review, we discuss recent progress in our understanding of eosinophil homeostatic activities within tissues, with particular emphasis on their functions in the GI tract, the largest reservoir of tissue-resident eosinophil in the body. As most available data related to the study of intestinal eosinophils are derived from animal experiments, we primarily focus here on the mouse. We briefly summarize their recently described functions within different tissues under baseline conditions and highlight environmental signals emerging as key regulators of their activities. We further discuss evidence of eosinophil transcriptional and functional heterogeneity in diverse tissue compartments, in light of new studies proposing developmental plasticity and tissue adaptation as major drivers of their polarization into functional subtypes.

### Homeostatic functions of tissue-resident eosinophils

**Figure 1.**
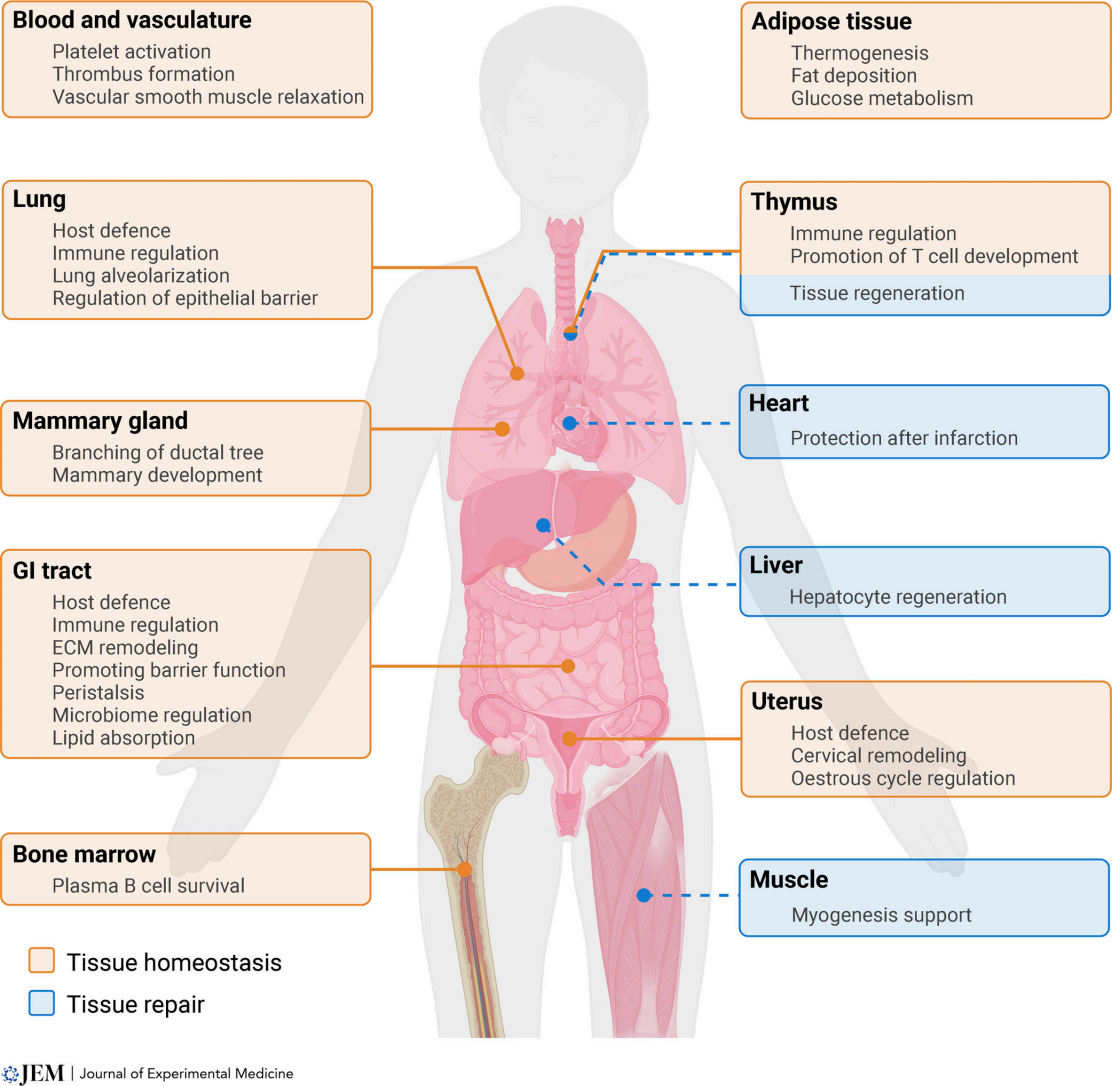
**Eosinophil functions in tissue homeostasis and repair.** In organs of residency, eosinophils contribute to tissue integrity and homeostasis (orange). Following tissue damage, eosinophils can be recruited to organs where they are not typically present and participate in tissue repair (blue).

As initially postulated by James J. Lee and co-workers in their provocative “LIAR hypothesis” (eosinophils as regulators of Local Immunity And/or Remodeling/Repair), eosinophils are often found at sites undergoing high epithelial turnover, remodeling, or stem cell activity, suggesting they might be inherently associated with the regulation of these processes, both in health and disease (Lee et al., 2010). More than a decade later, multiple reports have confirmed the homeostatic activities of eosinophils in various tissues, mostly supported by data from animal models of constitutive or inducible eosinophil deficiency, or models of conditional gene ablation targeting the eosinophil lineage. Below, we describe some of the functions of eosinophils that emerge as a common theme across tissues. While this list is not exhaustive, we refer the reader to the following excellent recent reviews for additional information (Shah et al., 2020; Jacobsen et al., 2021), including eosinophil activities in metabolic homeostasis, vascular tone, and neural health. An overview of eosinophil functions in healthy tissues and during tissue regeneration is shown in Fig. 1.

#### Tissue morphogenesis

Temporally controlled eosinophil recruitment waves are observed in several organs and are typically associated with stages of tissue development or morphogenesis. In the lungs, eosinophils are not present at birth but increase following the release of IL-5 by ILC2, in response to epithelial-derived IL-33 induced by the “first breath” (Saluzzo et al., 2017). Their postnatal recruitment peaks on days 10–13 and coincides with the lung alveolarization phase associated with the provisional deposition of extracellular matrix (ECM) components (Loffredo et al., 2020).

Eosinophils also seem to play an important role in the female mammary gland development and reproductive system. They are recruited to the growing terminal end buds of mammary glands in puberty to promote the branching of the ductal tree, in concert with macrophages (Gouon-Evans et al., 2000). They are again recruited during pregnancy when further mammary development occurs (Colbert et al., 2005). In IL-5–deficient mice, which lack most peripheral eosinophils, anatomical alterations and lower overall density of mammary gland structures have been reported. Their progeny was thus notably underweight upon nursing, with a high percentage of preweaning mortality (Colbert et al., 2005). Eosinophils are also cyclically recruited to the endometrium of the uterus during the estrous cycle under the influence of estradiol-mediated eotaxin-1 release, and their numbers peak during the degradation cycle of estrus to metestrus. In the absence of eotaxin, mice displayed a delayed onset of estrous cycles and age of first parturition, suggesting a role for eosinophils in preparing the mature uterus for pregnancy (Gouon-Evans and Pollard, 2001). Eosinophils also accumulated and degranulated in the cervix during pregnancy and labor when tissue degradative and reparative processes occur (Knudsen et al., 1997). However, despite the apparent association of eosinophils with uterine remodeling activity, eosinophil-deficient mice exhibit normal pregnancy, parturition, or postpartum uterine repair, suggesting that their role in this process is redundant (Robertson et al., 2000; Gouon-Evans and Pollard, 2001).

#### Tissue regeneration

While eosinophils might exert subtle remodeling activities to maintain homeostasis in their tissues of residency, they also infiltrate highly regenerative organs in which they are not normally present to promote the repair of wounded tissues. This activity seems to primarily depend not only on their ability to secrete IL-4, which promotes the proliferation of hepatocytes in the damaged liver (Goh et al., 2013), but also stimulates the proliferation of muscle resident fibro/adipocyte progenitors to inhibit their differentiation into adipocytes following muscle injury (Heredia et al., 2013). In response to IL-4 and the cationic protein mEar1, eosinophils were further reported to protect against ischemic injury in the infarcted heart. Liu et al. (2020) showed that eosinophils rapidly accumulated in the blood and heart following myocardial infarction and were essential to reduce subsequent cardiac dysfunction, cardiomyocyte apoptosis, and fibrosis, as well as to limit the adhesion of inflammatory cells.

Eosinophils were also implicated in the restoration of thymic function during re-establishment of the adaptive immune system following ablative therapy (Cosway et al., 2022). Cosway and colleagues showed that eosinophil-deficient mice exhibited significantly reduced thymic size and weight than their wild-type counterparts on days 7 and 35 following sublethal total body irradiation. This was accompanied by an overall decrease of thymic cellularity, reduced numbers of epithelial subsets, as well as lower proportions of CD4/CD8 thymocyte subsets. In the long term, reduced thymic T cell development impaired the recovery of the peripheral αβT-cell pool, as alterations were still visible more than 3 mo following injury. Interestingly, eosinophil-mediated thymic regenerative response depended on the concerted action of natural killer T cell–dependent IL-4, which triggered eotaxin-1 production in the thymic stroma, and ILC2-dependent IL-5, indicating that the restoration of thymic function requires a complex network of innate immune cells (Cosway et al., 2022).

#### Maintenance of tissue architecture

Recent studies have shed light on the cardinal roles of eosinophils in maintaining the tissue architecture and physiological function of GI organs. As reported by Ignacio et al. (2022), ablation of the eosinophil lineage in Δdbl.GATA1 mice resulted in significant alteration of the small intestinal villous architecture, characterized by a reduced villous surface area, barrier leakage, and altered peristalsis, in conjunction with decreased lipid absorption. The absence of eosinophils primarily affected ECM components of the basement membrane, which forms an anatomical barrier between the epithelium and underlying connective tissue, subsequently altering epithelial cell turnover and barrier permeability. Eosinophil deficiency also altered intestinal motility and transit time, potentially by affecting the function of intestinal neuronal/glial cells. Indeed, lamina propria eosinophils were found close to stromal cells, including α-smooth actin-expressing myofibroblasts, glial cells, and neuronal axons (Ignacio et al., 2022). Similarly, Diny et al. (2022) reported that eosinophils upregulated multiple transcripts upon migration into the small intestine, which were related to ECM components as well as proteins functioning in ECM remodeling such as laminins, collagens, and matrix metalloproteinases. Interestingly, many of these genes depended on the expression of the Aryl hydrocarbon receptor (AHR), a ligand-activated transcription factor. AHR-deficient eosinophils were functionally impaired in their adhesion to ECM components and were less capable of degrading collagen derivatives (Diny et al., 2022), suggesting that eosinophils support the homeostatic remodeling and constitutive renewal of the ECM, a critical activity in the intestine where high cellular turnover rate and constant mechanical stress requires timely regeneration (Pompili et al., 2021).

#### Mucosal homeostasis and immune regulation

**Figure 2.**
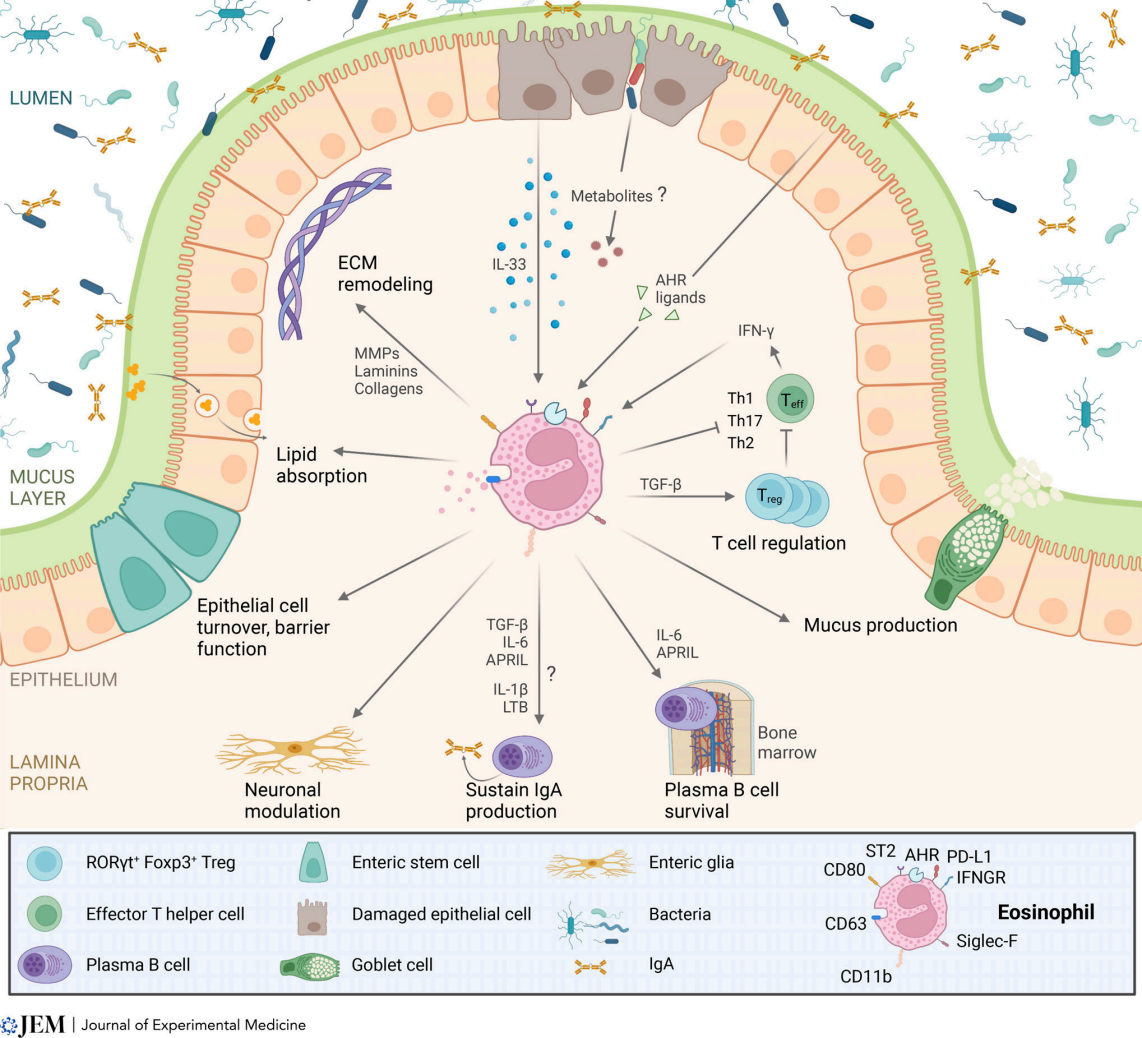
**Emerging functions of eosinophils in the GI tract.** In response to microbial and tissue-derived cues, intestinal eosinophils interact with the mucosal immune system, the epithelium, and microbiota to promote homeostasis. In the colon, eosinophils restrict Th1 responses against pathogens via upregulation of PD-L1, and eosinophil-derived TGF-β promotes the expansion of Foxp3^+^ Tregs. In the small intestine, eosinophils inhibit IL-17 baseline production by CD4^+^ T cells through the secretion of IL-1 receptor agonists and suppress Th2 responses during nematode infection. Eosinophils also release IL-6 and APRIL, which support plasma B cell survival in the bone marrow, and, together with IL-1β and lymphotoxin β (LTB), promote the generation and maintenance of IgA-producing plasma B cells in the small intestine. They further regulate GI motility via neuronal/glial cells and interact with non-immune cell types to maintain epithelial barrier integrity by enhancing mucus secretion and promoting epithelial cell migration. They also support the homeostatic remodeling and constitutive renewal of the ECM through the secretion of collagen, laminins, and the activity of matrix metalloproteinases (MMPs).

At mucosal sites, homeostasis depends on complex interactions between the microbiota, the intestinal epithelium, and the host immune system. This interplay is particularly important in the GI tract, where the layered immune system acts in concert with epithelial and stromal cell populations to mount effector responses against pathogens, while avoiding deleterious responses to the diverse commensal species of the microbiota. The balance between T cell effector and regulatory responses (1), the integrity of the epithelial barrier (2), as well as the presence of healthy microbial communities (3) contribute to ensuring mutualistic host–microbiota interactions, which are essential to maintain homeostasis. Interestingly, eosinophils have been implicated in the regulation of all these processes. A summary of their immunoregulatory and homeostatic activities in the GI tract is shown in Fig. 2.

*Regulation of the T cell compartment.* Increasing evidence suggests a role for eosinophils in regulating T cells by down-modulating T helper (Th) responses or supporting the differentiation of regulatory T cells (Treg) expressing the forkhead box P3 (Foxp3) protein. Eosinophils were reported to control the baseline production of IL-17 by CD4^+^ T cells in the small intestine through the constitutive secretion of IL-1 receptor antagonist, an inhibitor of IL-1β (Sugawara et al., 2016). Similarly, eosinophil depletion resulted in an increased frequency of colonic Th1 cells directed against components of the intestinal microbiota at steady-state and further failed to restrict excessive Th1 responses against the gastric pathogen *Helicobacter pylori* (Arnold et al., 2018). The control of Th1 responses required cell-intrinsic IFN-γ signaling in eosinophils, which led to the upregulation of PD-L1. In addition, PD-L1–expressing eosinophils accumulated in the colon of Crohn’s disease patients, where they localized in proximity to CD4^+^ T cells and showed evidence of an IFN-γ–associated signature (Gurtner et al., 2022), raising the possibility that IFN-γ–dependent PD-L1 expression might directly contribute to downmodulate Th1 response or might represent a useful marker for eosinophils with regulatory properties.

Eosinophils can also control adaptive T cell responses indirectly by promoting the expansion of Tregs. Intestinal, but not peripheral blood, eosinophils induced the differentiation of naive T cells into Foxp3^+^ Tregs cells in vitro through the release of TGF-β1 and retinoic acid (Chen et al., 2015). Furthermore, eosinophil-deficient mice display a notable reduction in the frequencies of intestinal Foxp3^+^ Tregs correlating with decreased TGF-β (Chu et al., 2014b). Interestingly, eosinophil-derived TGF-β was required for the expansion and tissue homing of a unique microbiota-dependent Treg subset, expressing the retinoic acid receptor–related orphan nuclear receptor gamma (RORγt) in the intestinal lamina propria following bacterial or allergen challenge (Fallegger et al., 2022). This subset was also reduced in the intestines of mice lacking responsiveness to AHR ligands in the eosinophil compartment, along with reduced expression of matrix metalloproteinase-9 (Diny et al., 2022), an enzyme that cleaves latent TGF-β into its active form (Kobayashi et al., 2014).

*Promotion of epithelial barrier integrity.* The integrity of the intestinal epithelial barrier is essential to provide protection against microbial intruders, whilst ensuring the absorption of nutrients and water. While eosinophils have been reported to compromise epithelial integrity in several tissues during infection or chronic inflammation through the release of their tissue-damaging granular proteins (Katinios et al., 2020; Furuta et al., 2005; Brottman et al., 1996), other studies suggest they may also contribute to the maintenance of healthy barrier functions, particularly in the absence of overt inflammation. Indeed, the loss of eosinophils in response to high-fat diet coincides with intestinal permeability, resulting in “leaky gut syndrome,” which can be reverted by re-establishing original eosinophil levels upon reinstatement of normal diet (Johnson et al., 2015). Eosinophils were also reported to enhance mucus secretion by goblet cells, support the renewal of ECM components, and enhance epithelial cell migration (Jung et al., 2015; Ignacio et al., 2022), thus promoting intestinal barrier integrity.

*Regulation of IgA and microbial composition.* Secretory immunoglobulin A (SIgA) are essential regulators of microbiota composition and function (Huus et al., 2021). In return, the microbiota composition also greatly determines the levels of IgA through both immune stimulatory and IgA degradation processes (Bunker et al., 2019; Moon et al., 2015). IgA and microbiota composition are thus intrinsically related. Chu et al. first reported that eosinophils support the survival of plasma cells in the bone marrow as well as the generation and maintenance of IgA^+^ plasma B cells in intestinal lamina propria via the release of IL-6 and APRIL (Chu et al., 2011; Chu et al., 2014b). In the absence of eosinophils, mice displayed reduced levels of SIgA and less IgA adherence to fecal bacteria, along with alterations in microbial composition (Chu et al., 2014b). A reduction of SIgA and microbial alteration in eosinophil-deficient mice was also reported by Jung et al. (2015) but relied on an indirect regulatory mechanism implicating eosinophil-derived IL-1β and lymphotoxins.

In contrast, later studies found only modest or no differences in the numbers of IgA-secreting plasma cells in eosinophil-deficient mice, proposing that the use of mice from different vendors rather than littermate controls might have accounted for these initially observed differences (FitzPatrick et al., 2020; Singh et al., 2019; Beller et al., 2020). Indeed, germ-free (GF) eosinophil-deficient and wild-type mice did not show differences in microbiota composition nor IgA levels following microbial recolonization, suggesting that eosinophils might not influence IgA levels in the absence of microbial differences (Ignacio et al., 2022). Interestingly, the expression pattern of eosinophil-derived APRIL, a well-known regulator of plasma cell homeostasis, has recently been suggested to depend on microbial stimulation and to exhibit strong spatial heterogeneity along the GI tract (Sturm et al., 2023), thus potentially explaining some of the discrepancies observed between studies. Nevertheless, a report using littermate controls revealed that the absence of eosinophils specifically affected the composition of intestinal mucus-resident bacterial species but was independent of IgA (Singh et al., 2019), raising the possibility that alteration in these mucus-residing bacteria might depend on epithelium-derived antimicrobial peptides or factors secreted by eosinophils themselves. Whether eosinophil-derived antimicrobial or granular proteins contribute to maintain host-microbial mutualism, possibly by limiting bacterial overgrowth or imposing selective pressures on the microbiota, is currently unknown.

## Eosinophil heterogeneity and plasticity within tissues

To maintain homeostasis, immune cells have developed functional adaptation programs tailored to their niche’s requirements, which are often dictated by the tissue itself. While the notion that eosinophils have the capacity to adapt to specific microenvironments is gaining increasing appreciation, it has so far mostly been based on their functional and surface marker heterogeneity. Eosinophils can thus display significant phenotypic differences across diverse tissues, within distinct anatomical compartments of the same organ or even within localized tissue microdomains at the same site (Gurtner et al., 2021; Chu et al., 2014a; Wen et al., 2012; Xenakis et al., 2018). Phenotypic diversity is also observed between health and disease state. The best-known example to date comes from the lung, where tissue-resident eosinophils strongly differ from eosinophils recruited during asthmatic challenge, prompting the authors to propose the existence of distinct cellular subsets. While resident eosinophils localize in the parenchyma and present a ring-shaped nucleus, inflammatory eosinophils recruited to the peribronchial areas during allergen challenge are characterized as Siglec-F^hi^CD62L^−^CD101^hi^, have a segmented nucleus, and present a proinflammatory gene profile, suggesting both subsets might have opposing roles (Mesnil et al., 2016).

The phenotypic diversity and pleiotropic functions of eosinophils has raised questions about their lineage ontogeny and the existence of distinct functional sub-phenotypes. In the following section, we highlight recent studies indicating that eosinophil maturation is not restricted to their original bone marrow niche but is a continuous differentiation process resulting from their adaptation to distinct tissue microenvironments. We further discuss some of the microbial and tissue-derived signals that were reported to shape eosinophil functional properties in the GI tract.

### Tissue adaptation in the intestinal niche

The strategic location of intestinal eosinophils close to the mucosal surface and their expression of various microbe-associated molecular patterns indeed indicates they can sense and respond to microbial stimulation. Interestingly, studies in GF mice have highlighted the role of the microbiota in shaping the phenotype and density of local eosinophil populations. Eosinophils were more numerous in the intestines of GF than in specific pathogen–free animals and exhibited a striking reduction of cytoplasmic granule size and content (Jiménez-Saiz et al., 2020). Ignacio et al. (2022) further showed that the recolonization of GF mice with complex microbiota resulted in a shorter half-life of intestinal eosinophils, consistent with increased cellular activity and reduced survival. Eosinophils of GF mice displayed strongly reduced sombrero vesicles and empty vesicles along with increased levels of intracellular eosinophil peroxidase than mice reared under specific pathogen–free conditions, suggesting that the microbiota drives the activation and homeostatic degranulation of intestinal eosinophils. Notably, the recolonization of GF mice prompted the release of IL-33 from epithelial cells, whose signaling was required for eosinophil homeostatic activities, thus pointing to an indirect effect of the microbiota on eosinophils via epithelial-derived cytokines.

In addition, Diny et al. (2022) reported that eosinophils adapt to the small intestinal niche by adopting a transcriptional tissue residency program that is reflected by changes in the expression of over 13,000 genes relative to bone marrow eosinophils. Interestingly, this transcriptional reprogramming partly depended on AHR signaling, which controlled several cell-intrinsic properties such as degranulation and lifespan, and further promoted eosinophil interactions with ECM components. As several endogenous AHR ligands are produced from the breakdown of food components or tryptophan catabolism by intestinal commensals (Schanz et al., 2020; Zelante et al., 2013), the selective activation of this pathway in intestinal eosinophils suggests their tissue specialization might be regulated by microbial components or metabolites.

Recently, Gurtner et al. (2022) characterized the mouse eosinophil lineage at the single-cell level and investigated the presence of transcriptionally defined eosinophil subsets. This study revealed that eosinophils develop from their bone marrow progenitors as five transcriptionally distinct subpopulations across tissues ordered along consecutive developmental and maturation stages, thus reflecting adaptation to distinct niches. The GI tract further harbored two distinct eosinophil subsets—an active (A-Eos) and a basal (B-Eos) population—differing in their transcriptome, surface proteome, and spatial localization. B-Eos localized predominantly at the bottom of crypts and displayed a typical Th2 profile characterized by STAT6 regulon activity as well as the synthesis of IL-4 and IL-13. They also upregulated transcripts such as TGF-β and the ECM-degrading enzyme matrix metalloproteinase-9. In contrast, A-Eos were found closer to the intestinal lumen and were characterized by enhanced secretory activity, antimicrobial potential, and proinflammatory cytokine profile (i.e., secretion of GM-CSF, TNF-α, and IL-1β). A-Eos further expressed several T cell co-stimulatory molecules and were characterized by the co-expression of CD80 and PD-L1, indicating they might be involved in the regulation of local T cell responses. Notably, challenge infection with bacterial pathogens or experimental colitis resulted in the strong accumulation of A-Eos at inflammation sites. Inflammation further promoted the acquisition of a robust antimicrobial gene signature in A-Eos, largely driven by local IFN-γ signaling, suggesting this subset might contribute to host defense.

Although both B- and A-Eos exhibited a non-overlapping transcriptional profile, trajectory analysis and subsequent in vivo validation experiments revealed that both subsets were inherently linked, with A-Eos arising from their less mature B-Eos counterparts, as a result of local tissue maturation driven by the cytokine IL-33 and microbial signals upstream of NF-κB and MAPK pathway signaling (Gurtner et al., 2022). In the GI tract, IL-33 is constitutively expressed from pericryptal fibroblasts and further increases during inflammation (Mahapatro et al., 2016). Interestingly, IL-33–stimulated eosinophils displayed a transcriptional profile similar to A-Eos and led to the upregulation of multiple cytokine receptors, including GM-CSFRα, IL-10Rα, as well as its own receptor ST2 (Gurtner et al., 2022). Therefore, IL-33 signaling may act as a checkpoint in the intestinal eosinophil tissue adaptation process, as it initiates a transcriptional cascade leading to the upregulation of new receptors, which in turn promotes responsiveness to local signals driving the functional specialization of A-Eos. In return, the cytokine network and selective ligand availability in diverse GI compartments may orchestrate regional specialization programs, leading to niche-specific functions.

Collectively, these studies demonstrate that eosinophils undergo extensive transcriptional remodeling and functional specialization upon intestinal residency, which are driven in response to the microbiota. Whether similar adaptation gene programs shape eosinophil activities at other mucosal sites is still unknown, yet likely.

### Plasticity of the eosinophil lineage

Evidence of eosinophil adaptation to distinct tissue niches suggests the highly plastic nature of this lineage. While local cues may shape tissue-specific functional properties under homeostatic conditions, environmental perturbations such as inflammation or allergen exposure may exert more profound alteration in the eosinophil lineage, already at early developmental stages. Eosinophils isolated from peripheral blood have thus often been shown to modulate surface receptor expression in the presence of systemic priming signals, such as IL-5 or IL-33 (Johansson et al., 2008; Johansson et al., 2020; Coppi et al., 2007; Gurtner et al., 2022). In their single-cell transcriptomic analysis, Gurtner et al. (2022) showed that challenge infection promoted the acquisition of novel inflammation-associated gene expression signatures, which were already upregulated in the bone marrow and blood. While this observation suggests that eosinophil functional programs are to a certain extent imprinted systemically in disease, it raises the question of whether and how this might affect the function of other eosinophil populations residing in distal organs. Indeed, a recent study showed that local allergic inflammation in the skin, gut, or lung strongly impacted the frequency and phenotype of tissue eosinophils within remote, allergen-nonexposed organs, which was associated with the priming of these organs for subsequent allergic inflammation (Olbrich et al., 2020). In contrast, the induction of experimental asthma promoted the resolution of arthritis in mice, and this protective effect was abolished upon eosinophil depletion (Andreev et al., 2020). Resolution of arthritis was associated with the emergence of a regulatory eosinophil subset expanding in response to IL-5 derived from lung ILC2 and was further present in the synovium of rheumatoid arthritis patients in remission, but not in active stage. Notably, in patients with rheumatoid arthritis and concomitant asthma, the depletion of eosinophil with an anti–IL-5 antibody (mepolizumab) induced relapse of arthritis in a majority of cases (Andreev et al., 2020). These observations suggest that eosinophil plasticity not only dictates their function in their tissue of residency but may also influence their activities during development, thus affecting their responses at distal sites.

## Concluding remarks

Recent advances in the field of eosinophil biology have shed light on the pleiotropic activities of these granulocytes in health and disease. The consensus that eosinophil functional diversity is driven by heterogenous responses to environmental cues is gaining increasing appreciation, mostly due to transcriptomic studies delineating eosinophil ontogeny, tissue adaptation, and functional specialization, along with animal studies investigating the relevance of eosinophil population in diverse tissues under non-inflammatory conditions. The study of GI eosinophils is particularly relevant in this regard, as they constitute the largest resident population of eosinophils in the body.

The evidence cited in this review supports the view that eosinophils actively contribute to the maintenance of intestinal homeostasis in response to microbial-derived signals, which drive a transcriptional cascade leading to their functional maturation. The existence of spatially compartmentalized transcriptional subsets in the GI tract may not be that surprising, given the steep hypoxic gradient and increasing microbial exposure from the submucosa to the lumen, as well as the selective presence of epithelial-derived cytokines at the luminal end, modulated by constant interaction with local microbial and immune networks. However, the signals that shape eosinophil functional properties at different stages of their tissue maturation are only beginning to be unraveled. A better understanding of the molecular pathways driving the differentiation of functional subsets in diverse organs is thus required to fully appreciate their contribution to local homeostatic and inflammatory processes. It may further lay the foundation for novel therapeutic approaches that rely on the rational modulation of eosinophil subsets in diverse clinical settings.
